# Venetoclax-based therapy for relapsed or refractory acute myeloid leukemia: latest updates from the 2023 ASH annual meeting

**DOI:** 10.1186/s40164-024-00486-7

**Published:** 2024-02-16

**Authors:** Xubo Gong, Xin He, Lin Wang, Teng Yu, Weiwei Liu, Huiying Xu, Lan Jin, Xiang Li, Bin Zhang, Zhihua Tao, Wenbin Qian

**Affiliations:** 1 Department of Clinical Laboratory, The Second Affiliated Hospital, Zhejiang University School of Medicine, Hangzhou, 310009 Zheijang P.R. China; 2 Department of Hematological Malignancies Translational Science, Gehr Family Center for Leukemia Research, Hematologic Malignancies and Stem Cell Transplantation Institute, Beckman Research Institute, City of Hope Medical Center, 1500 E Duarte Road, Duarte, CA 91010 USA; 3 Department of Hematology, The Second Affiliated Hospital, Zhejiang University School of Medicine, 88 Jiefang Road, Hangzhou, 310009 China

**Keywords:** Venetoclax, Acute myeloid leukemia, Hypomethylating agents

## Abstract

Patients with relapsed or refractory (R/R) acute myeloid leukemia (AML) often exhibit limited responses to traditional chemotherapy, resulting in poor prognosis. The combination of venetoclax (VEN) with hypomethylating agents has been established as the standard treatment for elderly or medically unfit AML patients unable to undergo intensive chemotherapy. Despite this, the availability of novel VEN-based therapies specifically tailored for those with R/R AML remains scarce. Here, we provide a comprehensive overview of the latest data presented at the 65th American Society of Hematology Annual Meeting, shedding light on the progress and efficacy of VEN-based therapies for R/R AML.

To the editor,

Relapsed or refractory (R/R) acute myeloid leukemia (AML) poses a formidable challenge with a grim prognosis. Venetoclax (VEN), a BCL2 inhibitor, has exhibited remarkable anti-leukemic activity in combination with hypomethylating agents (HMA) or low-dose cytarabine (Ara-C) for newly diagnosed unfit AML patients [1]. However, the efficacy of VEN-based therapy in R/R AML remains less defined. This review provides an overview of the recent advances in VEN-based therapies for R/R AML presented at the 2023 American Society of Hematology (ASH) meeting.

## VEN combined with chemotherapy drugs for R/R AML

VEN plus CPX-351, a liposomal fixed-ratio formulation containing Ara-C and daunorubicin, achieved a 45% overall response rate (ORR). After a median follow-up time of 20.7 months, the median overall survival (OS) and event-free survival (EFS) were 6.4 and 2.8 months, respectively. Among responders, the 2-year cumulative incidence of relapse and non-relapse mortality were 41% and 28%, respectively [2]. It produces encouraging response rates and is safe in R/R AML.

The clinical effectiveness of the VEN in combination with the CAG regimen (consisting of Ara-C, aclarubicin, and G-CSF) was assessed in patients diagnosed with R/R AML. The composite complete remission (CCR) was 78.4%, the rate of minimal residual disease (MRD) elimination in patients who achieved CCR was 44.8%, and the one-year OS was 78.4% [3]. It manifests that the combination is safe and active in patients with R/R AML.

The results of VEN plus high-dose Ara-C and mitoxantrone (HAM) in patients with R/R AML were updated. The mortality rates at 30 and 60 days were 2.6% and 5.2%, respectively. The complete remission (CR)/CR with incomplete count recovery (CRi) was 81.6%, and 21.9% of evaluable patients were MRD negative [4]. It confirms that VEN + HAM is a well-tolerated and very efficacious treatment option.

The initial efficacy of VEN and selinexor in combination with chemotherapy (fludarabine, Ara-C ± G-CSF) [FLA(G)] was assessed in pediatric, adolescent and young adult (AYA) patients with R/R AML. It demonstrated a CR/CRi rate of 41.7%, with 50% of the patients undergoing hematopoietic cell transplant following the completion of protocol therapy [5]. It shows that this combination is tolerable in AYA patients.

The investigation focused on studying the combination of VEN with liposomal mitoxantrone, homoharringtonine, and olverembatinib (MVHO), aiming to block multiple pathways in pediatric patients with R/R AML. After cycle one, the ORR was 94.4%, and the CR/CRi was 72.2%. The 8-month mean EFS was 60.1% and the OS was 100%. Throughout 26 cycles of MVHO treatment, there were no reported fatal infections or bleeding events [6].

In this series, VEN plus HAM seems to be effective and well-tolerated, while MVHO therapy may be a suitable treatment option for pediatric R/R AML, warranting further validation through larger clinical trials.

### VEN combined with epigenetic drugs for R/R AML

VEN with ASTX727 (oral decitabine/cedazuridine) in R/R AML achieved a 50% ORR. After a median follow-up of 12.8 months, the median OS was 7.6 months, with a median duration of response (DOR) of 4.6 months [7]. This combination is effective and tolerable.

The VEN, chidamide plus azacytidine (VAC) combination has exhibited synergy with both low-intensity and intensive chemotherapy in preclinical studies and in patients with R/R AML. The updated results of the VAC combination showed an ORR of 68%. With a median follow-up of 6.7 months, the median OS was not reached [8]. It proves that VCA has encouraging efficacy in patients with R/R AML.

A multi-center study was conducted to evaluate whether the addition of VEN to the azacitidine plus homoharringtonine (VAH) regimen could improve the response in patients with R/R AML. Compared with VEN + azacitidine, the triplet of VAH significantly improved composite remission rate (CRc) rate (66.3% vs. 44.3%, *P* < 0.001) and prolonged OS (no reach vs. 14.3 months, *P* = 0.004) in R/R AML [9].

Similarly, VAH plus sorafenib achieved a high ORR of 82.4% in R/R AML with *FLT3-ITD*. At a median follow-up of 10.3 months, the median OS was 18.1 months and EFS was 13.2 months [10].

In this series, an entirely oral regimen consisting VEN and ASTX727 may be an effective and convenient treatment option, while ‘VAH ± sorafenib’ regimes show high activity and promise, necessitating exploration in larger population trials.

### VEN combined with antibody or targeted drugs for R/R AML

VEN plus enasidenib, an oral selective IDH2 inhibitor, achieved a 70% ORR in *IDH2*-Mutated R/R AML. With a median follow-up of 17.1 months, the median OS was 9.4 months and the 2-year OS rate was 42%. Importantly, no cases of IDH inhibitor-associated differentiation syndrome were reported [11]. It shows that the combination is safe and well-tolerated in those patients.

The updated results of the combination of VEN and pegylated crisantaspase (PegC) showed an ORR of 47%, without serious asparaginase-related adverse events [12]. This combination is well-tolerated and can induce CR in some heavily R/R AML patients.

In conclusion, the 2023 ASH annual meeting showcased compelling efficacy and safety data for VEN-based therapies in R/R AML (refer to Tables 1 and 2), including MVHO, VAH ± sorafenib, VEN + enasidenib, etc. These findings underscore the feasibility of VEN-based treatments in managing R/R AML.

**Table 1:** VEN combined with chemotherapy drugs for R/R AML

Authors (reference)	Bataller [2]	Yu [3]	Ruhnke [4]	Zarnegar-Lumley [5]	Hu [6]
**Regimen**	VEN + CPX-351	VEN + CAG	VEN + HAM	VEN + selinexor + FLA(G)	VEN + MTO + HHT + olverembatinib
**Study type**	Phase Ib/II	N/A	Phase-I/II	Phase I	N/A
**NCT number**	NCT03629171	N/A	NCT04330820	NCT04898894	N/A
**Study period**	2018–2022	2016–2023	2020–2023	N/A	N/A
**Patients number**	33	37	38	14	18
**Male**	13 (39.4%)	24 (64.8%)	N/A	9 (64.3%)	9 (50.0%)
**Age range (years)**	26–72	18–68	26–74	3–17	0.3–13
**Median prior lines of therapies**	1 (1–7)	N/A	N/A	N/A	N/A
**Prior exposure to VEN**	19 (57.6%)	N/A	N/A	N/A	N/A
**Response rate**	ORR 45%,CR/CRi 39%	CRc 78.4%	CR/CRi 81.6%	CR/CRi 41.7	ORR 94.4%,CR/CRi 72.2%
**Grade ≥ 3 AEs**	Infections 45%	febrile neutropenia 67.6%	febrile neutropenia ≥ 10%	febrile neutropenia 16.7%	Neutropenia 100%
**Follow-up (months)**	20.7	N/A	N/A	N/A	N/A
**Survival**	Median OS 6.4 months	The one-year OS 78.4%	N/A	N/A	The 8-month mean EFS 60.1% and the OS 100%

AE adverse events, *AML* acute myeloid leukemia, *CAG* Ara-C, aclarubicin, and G-CSF, *CR* complete remission, *CRc* composite complete remission, *CRi* CR with incomplete count recovery, *EFS* event-free survival, *FLA(G)* fludarabine, Ara-C ± G-CSF, *HAM* high-dose Ara-C and mitoxantrone, *HHT* homoharringtonine, *MTO* mitoxantrone, *N/A* not available, *OS* overall survival, *ORR* overall response rate, *R/R* relapsed or refractory, *VEN* venetoclax

**Table 2:** VEN combined with epigenetic, antibody or targeted drugs for R/R AML

Authors (reference)	Bazinet [7]	Zha [8]	Yu [9]	Yu [10]	Richard-Carpentier [11]	Liu [12]
**Regimen**	VEN + ASTX727	VEN + AZA + chidamide	VEN + AZA + HHT	VEN + AZA + HHT + sorafenib	VEN + enasidenib	VEN + PegC
**Study type**	Phase II	Phase II	Retro	Phase II	Phase Ib/II	Phase I
**NCT number**	NCT04746235	N/A	N/A	N/A	NCT04092179	NCT04666649
**Study period**	N/A	2022–2023	2018–2022	2020–2022	2020–2022	N/A
**Patients number**	10	53	172	51	23	26
**Male**	N/A	31 (58.5%)	N/A	N/A	16 (59.3%)	10 (38.5%)
**Age range (years)**	46–75	18–73	N/A	31–57	23–84	24–79
**Median prior lines of therapies**	2 (1–4)	N/A	N/A	N/A	1 (1–2)	2 (1–5)
**Prior exposure to VEN**	N/A	N/A	N/A	N/A	N/A	18 (69.2%)
**Response rate**	ORR 50%, CR/CRi 50%	ORR 68%, CRc 53%	CRc 66.3%	ORR 82.4%, CRc 76.5%	ORR 70%, CR 57%	ORR 47%
**Grade ≥ 3 AEs**	Neutropenic fever 23%	No	N/A	Neutropenia 92.2%	febrile neutropenia 41%	N/A
**Follow-up (months)**	12.8	6.7	N/A	10.3	17.1	N/A
**Survival**	Median OS 7.6 months, median RFS or DOR 4.6 months	Median EFS 6 months, Median OS not reached	Median OS not reached	Median OS 18.1 months, EFS 13.2 months	Median OS 9.4 months, the 2-year OS rate 42%	N/A

*AE* adverse events, *AML* acute myeloid leukemia, *AZA* azacitidine, *CR* complete remission, *CRc* composite complete remission, *CRi* CR with incomplete count recovery, *DOR* duration of response, *EFS* event-free survival, *HHT* homoharringtonine, *N/A* not available, *OS* overall survival, *ORR* overall response rate, *PegC* pegcrisantastase, *R/R* relapsed or refractory, *Retro* retrospective, *RFS* relapse free survival, *VEN* venetoclax

## Data Availability

Not applicable.

## Abbreviations

AE: adverse events
AML: acute myeloid leukemia
AZA: azacitidine
CAG: Ara-C, aclarubicin, and G-CSF
CCR: composite complete remission
CR: complete remission
CRc: composite remission rate
CRi: CR with incomplete count recovery
DOR: duration of response
EFS: event-free survival
FLA(G): fludarabine, Ara-C ± G-CSF
HAM: high-dose Ara-C and mitoxantrone
HHT: Homoharringtonine
HMA: hypomethylating agents
MRD: minimal residual disease
ORR: overall response rate
OS: overall survival
PegC: pegcrisantastase
R/R: relapsed or refractory
VEN: venetoclax
